# The Negative Impact of Maternal HIV Infection on Birth Outcomes—Myth or Reality?

**DOI:** 10.3390/pathogens13090808

**Published:** 2024-09-18

**Authors:** Tudor Fleșeriu, Lorena Elena Meliț, Cristina Oana Mărginean, Anca-Meda Văsieșiu

**Affiliations:** 1 Department of Infectious Diseases, George Emil Palade University of Medicine, Pharmacy, Science and Technology of Targu Mures, 540136 Targu Mures, Romania; tudor.fleseriu@umfst.ro (T.F.); anca-meda.vasiesiu@umfst.ro (A.-M.V.); 2 Doctoral School of Medicine and Pharmacy, George Emil Palade University of Medicine, Pharmacy, Science and Technology of Targu Mures, 540136 Targu Mures, Romania; 3 Department of Pediatrics 1, George Emil Palade University of Medicine, Pharmacy, Science and Technology of Targu Mures, 540136 Targu Mures, Romania; marginean.oana@gmail.com

**Keywords:** mothers, HIV infection, birth outcomes, HEU

## Abstract

Human Immunodeficiency Virus (HIV) infection during pregnancy poses significant risks to both maternal and child health, with potential adverse effects on perinatal outcomes. This study aimed to compare perinatal outcomes, including birth weight, length, Apgar scores, and prematurity rates, between HIV-exposed, uninfected (HEU) children and HIV-unexposed, uninfected (HUU) children. A total of 204 neonates were included in the study, comprising 102 born to HIV-positive mothers and 102 born to uninfected mothers. Our findings revealed significant differences in birth weight (*p* < 0.001), length (*p* < 0.001), and Apgar scores at both 1 min (*p* = 0.003) and 5 min (*p* < 0.001) between HIV-exposed and -unexposed children. The HIV-exposed group exhibited lower birth weights and lengths, along with lower Apgar scores, indicating potential neonatal health challenges. No significant disparities were observed in the prematurity risk between the two groups (OR = 2.58, *p* = 0.126), but the risk of being born small for gestational age (SGA) in the case of HEU newborns was significantly high (OR = 17.41, *p* < 0.001). The significant differences in birth weight, length, and Apgar scores underscore the need for tailored healthcare interventions and support for neonates born to HIV-positive mothers. These findings contribute to our understanding of the complex interplay between maternal HIV infection and perinatal outcomes, guiding healthcare professionals in delivering targeted care for this vulnerable population.

## 1. Introduction

HIV infection remains a global health burden with major impact on maternal and child’s health. Despite substantial progress in the prevention and treatment of HIV over the last decades, the virus still affects 39 million individuals worldwide [1]. A great importance is given to fertile females infected with HIV, who represent the vast majority within the HIV-infected women population. The understanding of mother-to-child transmission (MTCT), HIV exposure, and their subsequent impact on birth outcomes is of paramount importance within this demographic.

HIV infection represents a great threat for the mother–offspring couple, the first major risk being the MTCT of the infection itself, which usually occurs during childbirth but can also take place through breastfeeding and, rarely, intrapartum [2]. MTCT was proven to range between 15% and 45% in the lack of medical intervention [3]. Secondly, due to the immune suppression induced by the virus, the mother is at an increased risk of contracting various infections, including opportunistic pathogens, some of which can have consequences not only on the maternal health status, but also on the child’s wellbeing [4,5]. There is also increasing evidence pointing out the fact that HIV-infected pregnant women are at a higher risk of developing pregnancy-related complications like preeclampsia, gestational diabetes mellitus, and premature rapture of membranes, besides having up to eight times higher mortality rates when compared to their uninfected counterparts [6,7]. HIV infection has also been correlated with complications involving the fetus, including miscarriages, stillbirths, preterm birth, and low birth weight [8,9,10]. Moreover, babies perinatally exposed to HIV, even if uninfected, seem to be at a higher risk of poorer overall growth, immune dysfunction, infectious morbidity, neurodevelopmental impairment, and mortality, when compared to the unexposed demographic [5,11,12].

The introduction of antiretroviral therapy (ART) improved considerably the prognosis of HIV-infected individuals, allowing many women living with HIV to minimize the infection’s effects on pregnancy. Moreover, ART alongside other prevention methods of mother-to-child transmission (PMTCT)-associated practices (HIV early diagnosis and monitoring, caesarian delivery, weaning while having detectable viral loads) have reduced the instances of newborn perinatal infection below 1% under optimal conditions [13,14]. Even under these circumstances, studies have shown that both maternal HIV infection and some ART molecules can influence the birth outcomes, potentially leading to adverse perinatal complications [8,15,16]. Thus, some studies associated certain antiretroviral agents with an increased risk of adverse perinatal outcomes including preterm birth, low birth weight, birth defects, preeclampsia, and even fetal death [17,18,19,20,21]. However, there are many studies highlighting the safety of ART use during pregnancy that were not able to correlate the aforementioned complications with HIV regimens when studied on a broader scale [22,23,24]. Taking into account the current controversial results reported in the aforementioned studies regarding the impact of HIV and/or ART on pregnancy, it is of great importance for further studies like ours to clarify the difference between myth and reality in terms of this topic. Nevertheless, it is crucial to note that the benefits of ART in preventing MTCT of HIV outweigh these potential risks by far and it is imperative to further investigate the potential implications of various ART regimens on maternal and fetal health in order to minimize the potential adverse outcomes and adapt the current guidelines accordingly.

### The Romanian “Historic HIV Cohort” (RHC)

In 1985, Romania reported its first case of HIV, but under its then-communist regime, it suppressed negative information, making communication with the West limited. After the 1989 revolution, alarming data emerged: many children in state hospitals and orphanages were infected with HIV. Over half of Europe’s HIV-positive children were found in Romania. Initial testing found 10% of hospitalized children and over 50% of children in orphanages to be HIV-positive. Between the late 1980s and the early 1990s, around 13,000 children were infected. International assistance played a pivotal role in helping Romania and its HIV-infected citizens navigate these challenges [25]. In 2001, the Ministry of Health declared the infection a public health priority by developing a national plan for universal access to treatment and care for HIV. The widespread implementation of antiretroviral therapy in 1997–1998 and the achievement of universal access to this treatment beginning in 2002 were crucial developments [26]. Key milestones include the implementation of an HIV surveillance system, the allocation of special budgets for HIV, the development of regional HIV centers, the initiation of various programs to prevent MTCT, the categorization of HIV data, and universal access to ART. By 2013, treatment was accessible regardless of the CD4 count. Despite the devastating early days of the epidemic, over half of the Romanian Cohort children are alive today. However, for the continued well-being of HIV patients, it is crucial for Romania to learn from its past, focus on consistent policy updates, address healthcare challenges, and ensure a continuous improvement in survival rates and quality of life [25].

Moreover, a considerable part of this population was early diagnosed with HIV infection and was granted access to the first available antiretrovirals. Most of these molecules, however, were proven by older studies to be linked to various side effects of different magnitudes, ranging from temporary rashes to conditions like liver failure, lactic acidosis, pancreatitis, etc. [27,28].

Despite these extensive studies, there is still a lack of comprehensive research comparing perinatal and postnatal parameters between HIV-infected and uninfected mothers. Furthermore, geographical and ethnic differences may influence these outcomes, possibly because of the lack of homogeneity within study groups. Understanding the impact of HIV infection on birth parameters and perinatal outcomes is critical for optimizing care and informing evidence-based guidelines for managing HIV-positive pregnancies. It is imperative to assess whether HIV-positive mothers face an elevated risk of delivering infants with adverse birth outcomes when compared to their HIV-negative counterparts. Therefore, studying the perinatal outcomes within a population that meets the homogeneity criteria in terms of HIV infection age and ART complexity may be an ideal opportunity to bypass the aforementioned limitations.

Hence, this study aims to answer the question of whether maternal HIV infection, particularly a long-term infection, significantly impacts birth outcomes such as birth weight, Apgar scores, and gestational age, by comparing these parameters between a group of newborns from HIV-positive mothers and a matched control group of newborns from HIV-negative mothers.

## 2. Materials and Methods

### 2.1. Study Sample

We performed a retrospective observational study between 2007 and 2022. The data were gathered form evidence of two maternities and the regional HIV monitoring and treatment center in Mures county, Romania. The inclusion criteria for the study group consisted of women who were either known with HIV before becoming pregnant or diagnosed with HIV during pregnancy or labor and their children. The exclusion criteria for the study group were women who became HIV-infected after giving birth, as we could not consider their children as being HIV-exposed, had a complex medical history, or were unable to be control-matched and newborn cases where PMTCT failed (perinatal infection or HIV confirmation within the first 2 years of life). Neonatal/pediatric HIV was ruled out according to the National Institute of Infectious Diseases “Prof. Dr. Matei Balș” recommendations, namely, negative HIV-RNA within the first 0–14 days of life, at 2 months, 4–6 months, 12 months, and 18 months, usually followed by further yearly virological monitorization up to 5–7 y.o. ± at least one negative serology after a minimum 6 months of age.

The collected data for both pediatric groups were perinatal parameters and outcomes (e.g., birth weight, birth length, Apgar scores, gestational age at birth).

### 2.2. Ethical Statements

The study was approved by the Ethics Committee of the Emergency County Hospital Targu Mures, No. 25400/09.10.2023 and the Ethics Committee of the County Hospital Targu Mures, No. 16456/30.09.2023. The study was performed according to the principles of the Helsinki Declaration.

### 2.3. Data Analysis

In order to compare the means of gestational week when the children were born (GA), birth weight (BW), birth lengths (BL), and Apgar scores at 1 (A1) and 5 min (A5), Student’s *t*/Mann–Whitney tests were performed. For assessing the risks of prematurity and being born small for gestational age (SGA), Fisher’s exact test was applied on contingency tables, followed by ROC analysis in order to quantify the predictive value that the mother’s seropositive status poses on these risks. We also tried to establish a correlation between prematurity, SGA risk, viral load, CD4 count, and the usage of protease and/or integrase inhibitors via Pearson 2-tailed test. While evaluating the impairment on perinatal outcomes attributed to a long, almost lifelong infection, the outcomes of the children born to RHC mothers were assessed separately, by comparing the same parameters to those of both their uninfected counterparts and children born to mothers who contracted the infection later in life. We defined newborns born before full 37 weeks of gestation as premature, and those falling in the SGA category were identified by using the international World Health Organization (WHO) growth charts. We used the software Microsoft Office Excel Version 2010, IBM-SPSS Statistics Version 20.0, Graph-Pad InStat Version 3.06.

## 3. Results

The study included 188 mothers and their 204 neonates, who were divided into two equal groups depending on the maternal HIV status (positive or negative), meaning 102 perinatally HIV-exposed, uninfected children (HEU) born to 86 HIV-infected women, of which 60 infants (HEUc) belonged to 48 RHC mothers, and 102 unexposed, uninfected children (HUU) in the control group born to 102 uninfected mothers. In order to strengthen the relevance of the results, given the small number of participants, the mothers in the two groups were age-matched (HIV mothers, mean age 25.5 y.o., min 16, max 39, SD: 0.49; non-HIV mothers, mean age 25.58 y.o., min 15, max 38, SD: 5.33). The mean age of the mothers included in the study did not differ significantly between the two groups (HEU vs. HUU *p* = 0.42; HEUc vs. HUU *p* = 0.81).

It is worth mentioning that out of the 93 registered births where the delivery type was specified, 90 were through C-sections (mean GA of 38.24 weeks), and 3 were natural births (mean GA of 38.25 weeks), of which two mother–baby pairs were lost to surveillance following maternity discharge, and one mother refused the PMTCT measures entirely. In the HIV-positive sample, 8 mothers tested positive for HIV during their pregnancy, 10 were detected as positive perinatally, and the remaining infected population already knew about their seropositive state when becoming pregnant. In the HIV-positive group, 11 mothers tested positive for hepatitis B surface antigen during their pregnancy screenings or at delivery and 1 had a confirmed hepatitis C infection.

From the available data, it appeared that the peripartum viral load was determined for only 78 births, of which 38 provided undetectable readings (<50 copies/mL). The remaining detectable 40 viral loads ranged from 68 to 975,116 (mean: 71.311) copies/mL. Finally, the mean viral load was 95,812 copies/mL in the RHC group and 25,955 copies/mL in the non-RHC group. The CD4 count was performed for 80 births and ranged from 30 to 1352 cells/mm^3^, with a mean of 545.7 cells/mm^3^ (RHC mean 487 cells/mm^3^ and non-RHC mean 663 cells/mm^3^).

The number of mothers falling in various stages of disease at birth according to the Centers of Disease Control and Prevention (CDC) classification is illustrated in Table 1. Of the 102 births present in our registries, only 78 had their HIV stage mentioned, and 24 lacked it.

Since most of the babies were born to RHC mothers, many of them had a long and complex treatment history. The general mean number of treatment regimens in each patient’s history was 3.01 (4.09 in the RHC group and 1.46 in the non-RHC group). In total, 56 of the ART schemes administered around the time of delivery included protease inhibitors (PI) like Lopinavir/Ritonavir (31), Atazanavir/Ritonavir (9), Darunavir/Ritonavir (13), or Saquinavir/Ritonavir (3), and 29 of them were integrase inhibitor (INSTI)-based (Raltegravir).

None of the uninfected mothers had severe documented comorbidities.

### Descriptive Analysis

Within the HEU group, a slight predominance of female newborns was observed (M:F = 47:55), whereas within the HUU group, males predominated (M:F = 60:42). Population characteristics revolving around the registered perinatal parameters within the studied populations are shown in Table 2.

**Figure 1:**
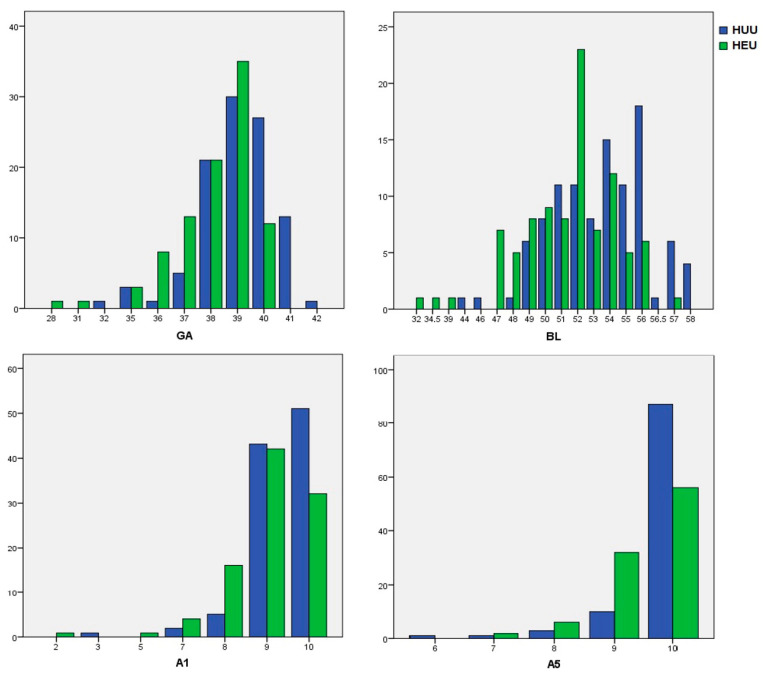
Back-to-back comparisons of GA, BL, and A1 and A5 (X axis: gestational weeks/centimeters/Apgar points; Y axis: number of neonates).

**Figure 2:**
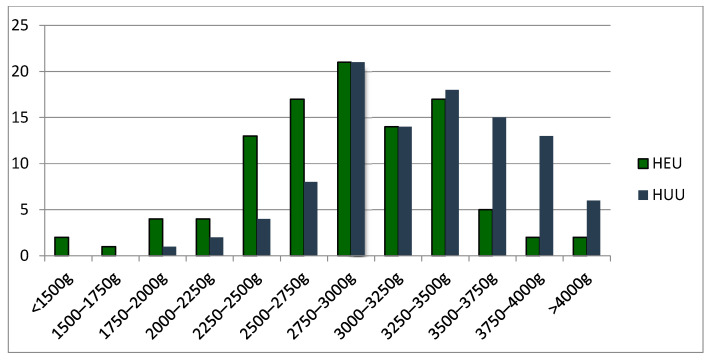
BW split in intervals.

Figure 1 shows back-to-back comparisons of GA, BL, and A1 and A5, respectively, while Figure 2 illustrates the BW split in intervals.

All of the compared parameters differed significantly. The most significant discrepancies regarded birth weight, with HEU newborns having a much lower mean birth weight (2885.9 g, 95% CI: 2776.7–2995 g) than their unexposed counterparts (3273.2 g, 95% CI: 3172.9–3373.6 g), *p* < 0.001. The newborns of the HEU group were also proven to be at a much higher risk of being born SGA (OR = 17.41, 95% CI: 2.25–134.59, *p* < 0.001). The HEU newborns were delivered slightly earlier than the children within the HUU group—GA mean 38.21 weeks (95% CI: 37.86–38.56 weeks) vs. 39.03 weeks (95% CI: 38.73–39.33 weeks), *p* < 0.001—but no higher risk of prematurity was identified (OR = 2.58, 95% CI: 0.87–7.63, *p* = 0.126). Regarding BL, the HEU newborns were generally shorter at birth, with a mean length of 51.26 cm (95% CI: 50.52–52.01 cm) than the newborns within the HUU group, whose mean BL was 53.35 cm (95% CI: 52.80–53.90 cm), *p* < 0.001. Finally, the Apgar scores tended to be slightly smaller within the HEU group, with a mean of 9.02 (95% CI: 8.78–9.26) at 1 min and of 9.52 (95% CI: 9.37–9.66) at 5 min versus 9.35 (95% CI: 9.17–9.54) at 1 min and 9.77 (95% CI: 9.65–9.90) at 5 min for the HUU group (A1: *p* = 0.003, A5: *p* < 0.001).

The HEU group was split in two groups depending on weather the mothers belonged to the RHC or not in order to investigate if a long-lasting infection and a complex ART history might be related to a further impairment of the investigated parameters. Therefore, the parameters of the 60 newborns belonging to RHC mothers (HEUc) were compared to those of the control group.

Once again, all of the investigated parameters were proven do differ significantly, and BW stood out as the most different one, with a mean of 2913.9 g (95% CI: 2763.1–3064.7 g) in the HEUc group vs. 3273.2 g (95% CI: 3172.9–3373.6 g) in the HUU group. The risk of being born SGA was proven to be even higher than in the previous comparison (OR = 20.20, 95% CI: 2.51–162.32, *p* < 0.001). The mean GA at birth within the HEUc group was once more noticeably different than that within the HUU group, with 37.97 weeks (95% CI: 37.16–38.78 weeks) vs. 39.03 weeks (95% CI: 38.73–39.33 weeks), (*p* < 0.001), but still no significant higher risk of prematurity was identified within this population (OR = 2.56, 95% CI: 0.77–8.47, *p* = 0.129). The HEUc median BL was slightly smaller, i.e., 51.62 cm (95% CI: 50.88–52.36 cm), but significantly different from the HUU median, which was 53.35 cm (95% CI: 52.80–53.90 cm), *p* < 0.001. The least-notable, but still significant, differences were found regarding A1 and A5: HEUc median A1 of 9.00 (95% CI: 8.65–9.35) vs. HUU median A1 of 9.35 (95% CI: 9.17–9.54), *p* = 0.02; and HEUc median A5 of 9.51 (95% CI: 9.31–9.71) vs. HUU median A5 of 9.77 (95% CI: 9.65–9.90), *p* < 0.001.

Finally, in order to investigate if a long exposure to the virus as well as a prolonged and complex ART history led to different perinatal outcomes, we separately compared the results of the 60 HEUc newborns to those of the remaining 42 HEU newborns, whose mothers had sexually contracted the infection later in life (HEUnc). The mean delivery time for the RHC mothers was 37.97 weeks (95% CI: 37.16–38.78 weeks), slightly shorter than that of the non-RHC mothers, which was 38.32 weeks (95% CI: 37.96–38.69 weeks), but without a significant difference (*p* = 0.7). Regarding BW, the HEUc newborns had a slightly larger BW, with a mean of 2913.9 g (95% CI: 2763.1–3064.7 g), than the HEUnc newborns, whose mean BW was 2840.3 g (95% CI: 2658.2–3022.4 g), but with no significant difference (*p* = 0.91). Similarly, we found a slightly greater BL for the HEUc newborns than for the HEUnc ones, with a mean of 51.62 cm (95% CI: 50.88–52.36 cm) versus 50.59 cm (95% CI: 48.88–52.50 cm), but without statistical significance (*p* = 0.23). Lastly, we found minor differences regarding the Apgar score, with a value of 9.0 (95% CI: 8.65–9.35) in the HEUc group vs. a value of 9.03 (95% CI: 8.69–9.38) in the HEUnc group at 1 min, and values of 9.51 in the HEUc group (95% CI: 9.31–9.71) and 9.55 in the HEUnc one (95% CI: 9.31–9.79) at 5 min, once again, without statistical significance (A1: *p* = 0.73, A5: *p* = 0.88).

In order to quantify the risk that the mother’s HIV seropositivity poses on the newborn’s outcomes, ROC curves were used, showing a good and significant prediction for neonates to be born SGA, with an AUC of 0.737 (95% CI: 0.638–0.837), *p* = 0.002, but without attaining statistical relevance in terms of prematurity prediction (*p* = 0.12).

Via the Pearson two-tailed correlation test, we managed to establish a positive correlation between being at the risk of being born SGA and PI usage (r = 0.276; *p* = 0.015), a negative correlation between the former parameter and INSTI usage (r = −0.255; *p* = 0.025), as well as a positive correlation between the same measure and the viral load (r = 0.362; *p* = 0.002), but no correlation with the CD4 count. No correlations were established between prematurity, viral load, CD4 count, and the studied highly active antiretroviral therapy (HAART) molecules.

## 4. Discussion

It is already a clearly established fact that HIV infection is responsible for a chronic, subtle inflammatory response, even if the viral load is persistently suppressed [29,30], as well as that chronic inflammation can have consequences on the outcomes of pregnancy. It is suggested that in the presence of abnormal levels of inflammatory mediators, nutrients and oxygen delivery may be impaired, resulting in overall fetal growth dysregulation [31,32,33]. There is large evidence highlighting the fact that there is a direct correlation between a chronic HIV infection and the impairment of perinatal outcomes. Moreover, HAART, especially protease inhibitors, usage has also been linked to a variety of possible adverse effects on pregnancy [18,34,35]. It was suggested that protease inhibitors might decrease the progesterone levels, increasing the risk of fetal growth restriction [36]. Another potential mechanism involved in the adverse birth outcomes related to this class of ART was related to decreased CYP450 enzyme activity and the subsequent lower levels of CYP4A and CYP2B6 eicosanoids in infants exposed to HIV and anti-retroviral therapy [37]. Nucleoside reverse-transcriptase inhibitors might also be associated with adverse pregnancy outcomes due to their negative effect on the mitochondrial DNA genome, resulting in mitochondrial dysfunction [38].

However, there is not a clear consensus so far regarding the impact of maternal infection and treatment on neonatal outcomes. Thus, our findings are of major importance in clinical practice, since they clearly underline the importance of a particular approach in the management of HIV-positive pregnant women involving a close monitoring during pregnancy, with the assessment of maternal nutrition and maternal sanitation habits during pregnancy and compliance to ART, along with the implementation of strategies to improve these parameters for achieving the best outcome in HIV-exposed, uninfected newborns.

### 4.1. Prematurity

Prematurity refers to the condition of being born before the completion of the normal 37 to 42 weeks of gestation. A premature birth may result in the newborn having underdeveloped organs and an increased risk of health complications due to their immaturity.

The premature HEU newborn cases within our research, although numerically superior to those born to unexposed women (12:5), were not a proof of a higher risk of preterm births, as the statistical analysis employed showed no statistical significance. When comparing premature births within the RHC group to those inside the non-RHC group, the differences were once more insignificant (7:5). However, we unveiled a significant difference between the mean GA of HEU and HUU newborns. Comparing the percentage of prematurity cases from this study group (11.7%) to the European countries averages, it may also seem that HEU children could be at a higher risk of being born earlier than predicted [39]. Similarly to our results, a meta-analysis by Schmid et al. disproves the fact that HAART represent a supplementary risk for preterm birth [40]. Moreover, a recent study performed by Portwood et al. shows that children born to women with HIV using HAART have better overall perinatal outcomes than those born to women without any anti-HIV medication, but still worse outcomes than unexposed children [15].

However, many studies addressing the topic of prematurity in children born to women living with HIV agree that both HIV and HAART intrauterine exposures are cumulating risk factors for earlier births [4,8,41]. Nevertheless, existing data regarding the length of the infection and/or the HAART therapy duration in relation with the severity or frequency of the impaired outcomes are scarce. A study by Uthman et al. shows that women who started ART before conception were more likely to deliver prematurely than those who began ART after conception [42], a phenomenon that was also investigated and confirmed by Sibiude et al. [43]. Based on our results, we found no significant differences regarding the outcomes of children born to RHC mothers when compared to those of children born to mothers who became infected later in their lifetimes.

### 4.2. Birth Weight

Our study managed to establish a very significant association between BW impairment and HEU children, as well as a heightened risk of being born SGA, hinting at an overall intrauterine restriction of growth. It is worth mentioning that our control group only had one SGA newborn (0.9%), which is an extremely low percentage when compared to the European SGA incidence [44]. For all the compared parameters, the highest statistical significance was obtained while comparing the mean BW of HEU newborns to that of their unexposed peers, further suggesting an impairment of intrauterine growth.

Comparing the existing evidence to that regarding the topic of prematurity in HEU newborns, it seems to be a better established fact that this demographic usually has lower BW rates [9,18]. It is suggested that both HIV infection and ART have a combined effect, partially limiting the full somatic development of newborns [8,15]. Similarly to the impact on GA with respect to the ART time of initiation, women who started receiving their treatments during pregnancy were associated with a higher probability of delivering SGA newborns [41,42]. However, this study failed in proving a significant association between the severity of BW impairment and the length of the infection or ART treatment, since we found no significant differences when comparing the mean BW and the risks of SGA between HEUnc and HEUc children. Still, it is a well-documented fact that both prematurity and being born SGA are associated with a higher mortality rate in children, adding to the overall risks of HEU children [45,46,47]. Given these findings, it is imperative for HIV-positive pregnant women to be more closely monitored, ideally by a multidisciplinary team (obstetrician, specialist in infectious diseases, general practitioner, dietician) and according to a protocol especially designed for this at-risk population and, whenever possible, applying tailored interventions in order to prevent the potential well-documented adverse outcomes during the stressful period of pregnancy.

### 4.3. Apgar Score

This assessment helps medical professionals identify any immediate health concerns and determine if the newborn requires any urgent medical attention [48]. Lower Apgar scores have been linked to higher mortality and morbidity rates in neonates and may serve as a good prognostic tool [49]. Once more, the available data do not seem to be conclusive regarding whether the Apgar scores differ in relation to HIV maternal seropositivity, since most studies did not describe a significant difference in Apgar scores when comparing HEU to HUU children [50,51]. However, similarly to our article, some studies described a relevant difference between the two populations [52].

This study was limited by the small number of patients included in both groups and also by the fact that we did not assess the impact of anthropometric maternal parameters on newborn outcomes, since the maternal nutritional status might influence the neonatal anthropometric parameters. Although our study involved a carefully selected population in both groups with similar characteristics, given the small sample size, it is worth mentioning that the aforementioned results cannot be generalized with a high degree of certainty on a larger scale. In order to establish more accurate and generalizable results, larger, prospective studies must be conducted that should also take into account other parameters (e.g., maternal nutrition during pregnancy, hygiene habits) in order to avoid bias.

## 5. Conclusions

Based on the aforementioned results, we conclude that HEU children may have impaired perinatal outcomes, especially regarding their BW, suggesting that the subtle inflammatory state associated with the chronic infection and the in-utero exposure to some HAART regimens may be linked to an overall intrauterine growth limitation. Given that within the examined populations most babies were born earlier and during their Apgar evaluations received lower scores, they might have been prone to higher risks of morbidity and mortality. By studying the birth outcomes of RHC mothers, a particular population due to its long HIV infection and complex HAART history, we had the opportunity of investigating the cumulative effects of the infection on pregnancy. In this context, we could state that the length of maternal infection and HAART usage might be link to a higher risk of being born SGA. Nevertheless, further studies on larger samples are needed in order to precisely identify the impact of maternal HIV infection and HAART exposure on birth outcomes, taking into account other parameters that might be involved in impairing newborn wellbeing, as well as the development of multidisciplinary screening protocols and tailored interventions for both HIV-positive pregnant women (involving an obstetrician, a specialist in infectious diseases, a nutritionist) and HIV-exposed, uninfected children, particularly during the first years of life (involving a neonatologist, a pediatrician, a cardiologist, a neurologist, a psychologist, a nutritionist). Additionally, studies should also focus on correlating certain adverse outcomes to specific HAART molecules and develop new treatment recommendations for pregnant women according to their relevant findings.

## Figures and Tables

**Table 1:** Number of mothers falling in various stages of disease at birth according to CDC classification.

A1: 10	A2: 5	A3: 0
B1: 4	B2: 12	B3: 14
C1: 2	C2: 7	C3: 24

**Table 2:** Descriptive analysis of the perinatal parameters.

HEU (102)	GA (Weeks)	BW (g)	BL (cm)	A1	A5	HUU (102)	GA (Weeks)	BW (g)	BL (cm)	A1	A5
**Mean**	38.21	2885.9	51.26	9.02	9.52	**Mean**	39.03	3273.2	53.35	9.35	9.77
**95% CI**	37.86–38.56	2776.7–2995.0	50.52–52.01	8.78–9.26	9.37–9.66	**95% CI**	38.73–39.33	3172.9–3373.6	52.80–53.90	9.17–9.54	9.65–9.90
**SD**	1.643	512.1	3.5	1.13	0.68	**SD**	1.525	510.97	2.80	0.93	0.64
**Median**	39	2860.0	52	9	10	**Median**	39	3300	54.000	9.50	10
**MIN**	28	1150	32	2	7	**MIN**	32	1800	44	3	6
**MAX**	40	4160	57	10	10	**MAX**	42	4500	58	10	10
**HEUc (60)**	**GA (Weeks)**	**BW (g)**	**BL (cm)**	**A1**	**A5**	**HEUnc** **(42)**	**GA (Weeks)**	**BW (g)**	**BL (cm)**	**A1**	**A5**
**Mean**	37.97	2913.9	51.62	9	9.51	**Mean**	38.32	2840.3	50.69	9.03	9.55
**95% CI**	37.16–38.78	2763.1–3064.7	50.88–52.36	8.65– 9.35	9.31–9.71	**95% CI**	37.96–38.69	2658.2–3022.4	48.88–52.50	8.69– 9.38	9.31–9.79
**SD**	2.13	547.21	2.69	1.28	0.72	**SD**	1.32	478.78	4.73	0.90	0.63
**Median**	39	2900	52	9	10	**Median**	39	2800	52	9.00	10
**MIN**	28	1650	47	2	7	**MIN**	35	1150	32	7	8
**MAX**	40	4160	57	10	10	**MAX**	40	3510	55	10	10

Legend: HEU = HIV-exposed, uninfected; HEUc = HIV-exposed but uninfected born to mothers from RHC; HEUnc = HIV-exposed but uninfected born to mothers infected later in life; HUU = HIV-unexposed, uninfected; GA = gestational age at birth; BW = birth weight; BL = birth length; A1 = Apgar score at 1 min; A5 = Apgar score at 5 min; 95% CI = 95% confidence interval for mean; SD = standard deviation; MAX = maximum value; MIN = minimum value.

## Data Availability

The data presented in this study are available on request from the corresponding author.
